# TRIM66 overexpresssion contributes to osteosarcoma carcinogenesis and indicates poor survival outcome

**DOI:** 10.18632/oncotarget.4291

**Published:** 2015-06-17

**Authors:** Yu Chen, Yongfei Guo, Haisong Yang, Guodong Shi, Guohua Xu, Jiangang Shi, Yin Na, Deyu Chen

**Affiliations:** ^1^ Department of Orthopedic Surgery, Changzheng Hospital, Second Military Medical University, Shanghai 200003, China; ^2^ Department of Anesthesiology & Critical Care Medicine, Xin Hua Hospital Affiliated to Shanghai Jiao Tong University School of Medicine, Shanghai 200092, China

**Keywords:** TRIM66, osteosarcoma, p53, TGF-β, EMT

## Abstract

TRIM66 belongs to the family of tripartite motif (TRIM)-containing proteins. Alterations in TRIM proteins have been implicated in several malignancies. This study was aimed at elucidating the expression and biological function of TRIM66 in osteosarcoma. Here, TRIM66 expression level was higher in osteosarcoma tissues than in normal tissues. High TRIM66 expression was correlated with high rate of local recurrence and lung metastasis, and short survival time. Then, we found that knockdown of TRIM66 in two osteosarcoma cell lines, MG63 and HOS, significantly inhibited cell proliferation and induced G1-phase arrest. Moreover, inhibition of TRIM66 in osteosarcoma cells significantly induced cell apoptosis, while remarkably inhibited cell migration, invasion as well as tumorigenicity in nude mice. Gene set enrichment analysis in Gene Expression Omnibus dataset revealed that apoptosis, epithelial-mesenchymal transition (EMT) and transforming growth factor-β (TGF-β) signaling pathway-related genes were enriched in TRIM66 higher expression patients, which was confirmed by western blot analysis in osteosarcoma cells with TRIM66 silenced. In conclusion, TRIM66 may act as an oncogene through suppressing apoptosis pathway and promoting TGF-β signaling in osteosarcoma carcinogenesis. TRIM66 may be a prognostic factor and potential therapeutic target in osteosarcoma.

## INTRODUCTION

TRIM66 (also known as transcription intermediate factor 1δ, TIF1δ) is a member of the family of tripartite motif (TRIM)-containing proteins [1]. The TRIM family of proteins is defined by the presence of an N-terminal tripartite motif composed of a RING domain, 1 or 2 B-box motifs and a coiled-coil region. TRIM proteins are further classified into 12 subgroup on the basis of their various C-terminal domains. TRIM66, which contains a PHD domain followed by a BROMO domain at the C terminus, belongs to a subgroup, consisting of TRIM24, TRIM28, TRIM33 and TRIM66 [2]. Most of the TRIM family proteins could be defined as E3 ubiquitin ligases. Almost all oncogene products and tumor suppressors are regulated by post-translational modifications, including the ubiquitin-proteasome system [3]. Although little is known about the expression pattern and biological functions of TRIM66 in cancers, a number of studies have revealed that altered expression of other TRIM family members is associated with cancers and cancer-related diseases. For instance, TRIM24 expression has been reported to be upregulated in myelodysplastic syndrome-related acute myeloid leukemias [4] and breast cancer [5, 6]. Reduce expression of TRIM33 is found in chronic myelomonocytic leukaemia [7]. Upregulation of TRIM28 (also known as TIF1β) [8] and TRIM25 [9] is associated with a poor prognosis in gastric cancer and breast cancer, respectively.

Osteosarcoma derives from primitive bone-forming mesenchymal cells and is the most common primary bone malignancy in children and adolescents [10]. It can arise in any bone but predominantly in the metaphyses of long bones [11]. The development of combination treatment with radical surgery and neoadjuvant chemotherapy has significantly increased the survival rates from 20 to 75% [12, 13, 14]. However, a substantial number of patients still experience local recurrence or distant metastases after curative resection of their primary tumors. The clinical outcome is extremely poor for these recurrent or metastatic patients [15]. Consequently, new prognostic markers and effective therapeutic targets for osteosarcoma is urgent needed.

In the current study, we explored the expression and biological functions of TRIM66 in osteosarcoma and sought to identify the involved mechanisms. We found that the mRNA level of TRIM66 was significantly increased in osteosarcoma tissues compared to bone cysts tissues. Further clinical characteristics analysis showed that high expression level of TRIM66 was associated with local recurrence, lung metastasis and poor survival rate in patients with osteosarcoma. Then we explored the role of TRIM66 in osteosarcoma cells by RNA interference. We found that knockdown of TRIM66 inhibited cell proliferation, cell cycle progression and metastasis, but induced cell apoptosis. Furthermore, gene set enrichment analysis (GSEA) with Gene Expression Omnibus (GEO) E-MEXP-3628 dataset showed that TRIM66 was positive related with apoptosis, epithelial-mesenchymal transition (EMT) and transforming growth factor-β (TGF-β) signaling pathway, which was further confirmed in osteosarcoma cells with TRIM66 silenced. Collectively, our data suggest that TRIM66 is a potent prognostic factor of osteosarcoma and TRIM66 plays a critical role in osteosarcoma carcinogenesis.

## RESULTS

### The elevated level of TRIM66 was correlated with poor prognosis of osteosarcoma

**Figure 1:**
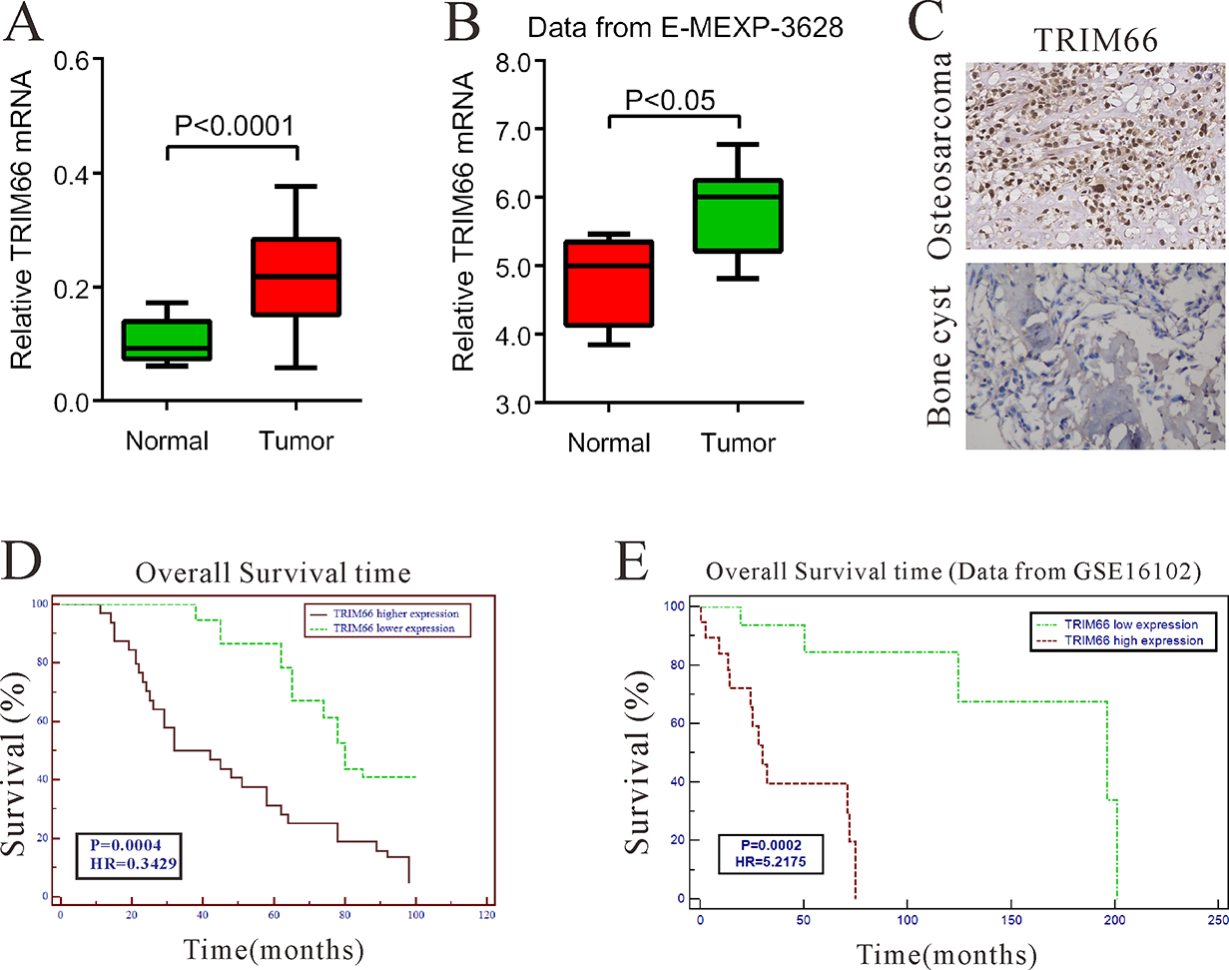
Correlation between TRIM66 expression and survival time of patients with osteosarcoma **A.** TRIM66 mRNA level was significantly higher in osteosarcoma tissues (*n* = 45) than that in bone cysts (*n* = 14) from patients treated at Department of Orthopedic Surgery, Changzheng Hospital (Shanghai, China) between 2000 and 2006 (*P* < 0.0001); **B.** TRIM66 expression was significantly increased in osteosarcoma tumor tissues (*n* = 14) when compared with normal bone tissues (*n* = 4) of patients from GEO dataset E-MEXP-3628 (*P* < 0.05); **C.** The protein level of TRIM66 by immunohistochemistry in osteosarcoma tissues and bone cysts; **D.** The overall survival time of 101 patients with osteosarcoma; **E**. Survival analysis of patients from ArrayExpress dataset GSE 16102.

To investigate the TRIM66 expression in osteosarcoma, we first detected its mRNA levels in 45 osteosarcoma tissues and 14 bone cysts by real-time PCR. Our data suggested TRIM66 was significantly overexpressed in osteosarcoma tissues compared with that in bone cysts (Figure 1A, *P* < 0.05), which was consistent with the analysis on data from GEO dataset (Access ID: GSE3628) (Figure 1B, *P* < 0.05).

Then, we further investigated the TRIM66 protein expression in osteosarcoma and bone cysts by immunohistochemistry. TRIM66 was up-regulated in 64 out of 101 (63.4%) tumor tissues compared with bone cysts (Figure 1C).

To investigate whether TRIM66 overexpression correlates with osteosarcoma prognosis, we analyzed the correlations between clinicopathological characteristics and the protein expression of TRIM66 in 101 patients with osteosarcoma by Chi-square test. TRIM66 expression positively correlated with local recurrence (*P* = 0.0009) and lung metastasis (*P* = 0.0003), although there was no significant relationship between TRIM66 expression and other factors, age, gender, tumor location or stage (Table 1). Kaplan-Meier survival analysis (Figure 1D) indicated that the survival time of patients with higher TRIM66 expression was significantly shorter than that of patients with lower TRIM66 expression (*P* < 0.001), which was further confirmed by survival analysis on GEO dataset (Access ID: GSE16102) (Figure 1D, *P* < 0.001). These results indicated that TRIM66 expression was elevated in osteosarcoma tissues, which was correlated with poor survival of osteosarcoma patients.

**Table 1:** Relationship between expression level of TRIM66 and clinical characteristics in osteosarcoma (*n* = 101)

		TRIM66 (*n* = 37) Negative and low		TRIM66 (*n* = 64) High		*P*-value
		*N*	%	*N*	%	
Gender	Male	21	56.8	29	45.3	0.3059
	Female	16	43.2	35	54.7	
Age at diagnosis	<14	26	70.3	36	56.3	0.2049
	> =14	11	29.7	28	43.8	
Site	Femur	24	64.9	34	53.1	0.2345
	Tibia	7	18.9	10	15.6	
	Fibula	1	2.7	0	0.0	
	Humerus	4	10.8	14	21.9	
	Others	1	2.7	6	9.4	
Stage grouping	Early	15	40.5	24	37.5	0.8332
	Advanced	22	59.5	40	62.5	
Local	No	25	67.6	21	32.8	0.0009^***^
Recurrence	Yes	12	32.4	43	67.2	
Lung metastasis	No	23	67.6	16	25.0	0.0003^***^
	Yes	14	32.4	48	75.0	

**Note:** *P* values are from chi-square test and were significant at <0.05.

*** *P* < 0.001.

### Silencing of TRIM66 expression by siRNA inhibited the proliferation of osteosarcoma cells

**Figure 2:**
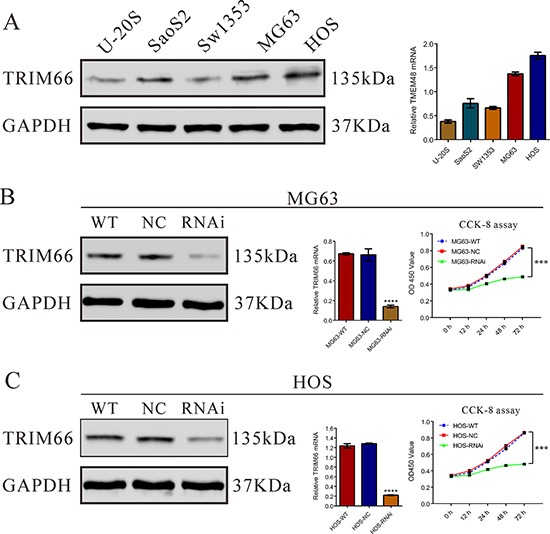
Depletion of TRIM66 inhibited cell growth in osteosarcoma cells **A.** TRIM66 expression level in 5 osteosarcoma cell lines was analyzed by real-time PCR (right panel) and Western blot (left panel). Data were based on at least three independent experiments; **B, C.** Expression of TRIM66 in MG63 cells and HOS cells was analyzed by real-time PCR (middle panel) and Western blot (left panel). Cell proliferation (right panel) was detected 12, 24, 48 and 72 hours after transfection in MG63 and HOS cells. Data were based on at least three independent experiments, and shown as mean ± S.D. WT: wild type cells; NC: scrambled siRNA-transfected cells; RNAi: TRIM66-siRNA-transfected cells (***P < 0.001 as compared with NC).

We then estimated TRIM66 expression in five osteosarcoma cell lines, U-20S, Saos2, Sw1353, MG63 and HOS, by real-time PCR and Western blot. As shown in Figure 2A, two cell lines, MG63 and HOS, showed higher mRNA and protein expression, were chosen for further study.

To investigate the function of TRIM66 in osteosarcoma, one siRNA targeting human TRIM66 (TRIM66-siRNA) and a non-specific scramble siRNA (NC) were synthesized to transfected MG63 and HOS cells. TRIM66-siRNA was able to efficiently suppress endogenous TRIM66 expression in osteosarcoma cells, whereas TRIM66 expression remained unaffected in NC-transfected cells (Figure 2B & 2C). The knockdown ratio was 79.1% and 82.1% in MG63 and HOS cells, respectively.

We then examined the proliferation of cells transfected with TRIM66-siRNA using CCK-8 assay. In MG63 and HOS cells with TRIM66 silenced, cell growth was significantly decreased at 24 h, 48 h or 72 h compared to scramble siRNA transfected cells (Figure 2B & 2C, *P* < 0.001). These results showed the anti-proliferation effect of TRIM66 siRNA in osteosarcoma cells.

### Silencing of TRIM66 induced G1 phase arrest and cell apoptosis

**Figure 3:**
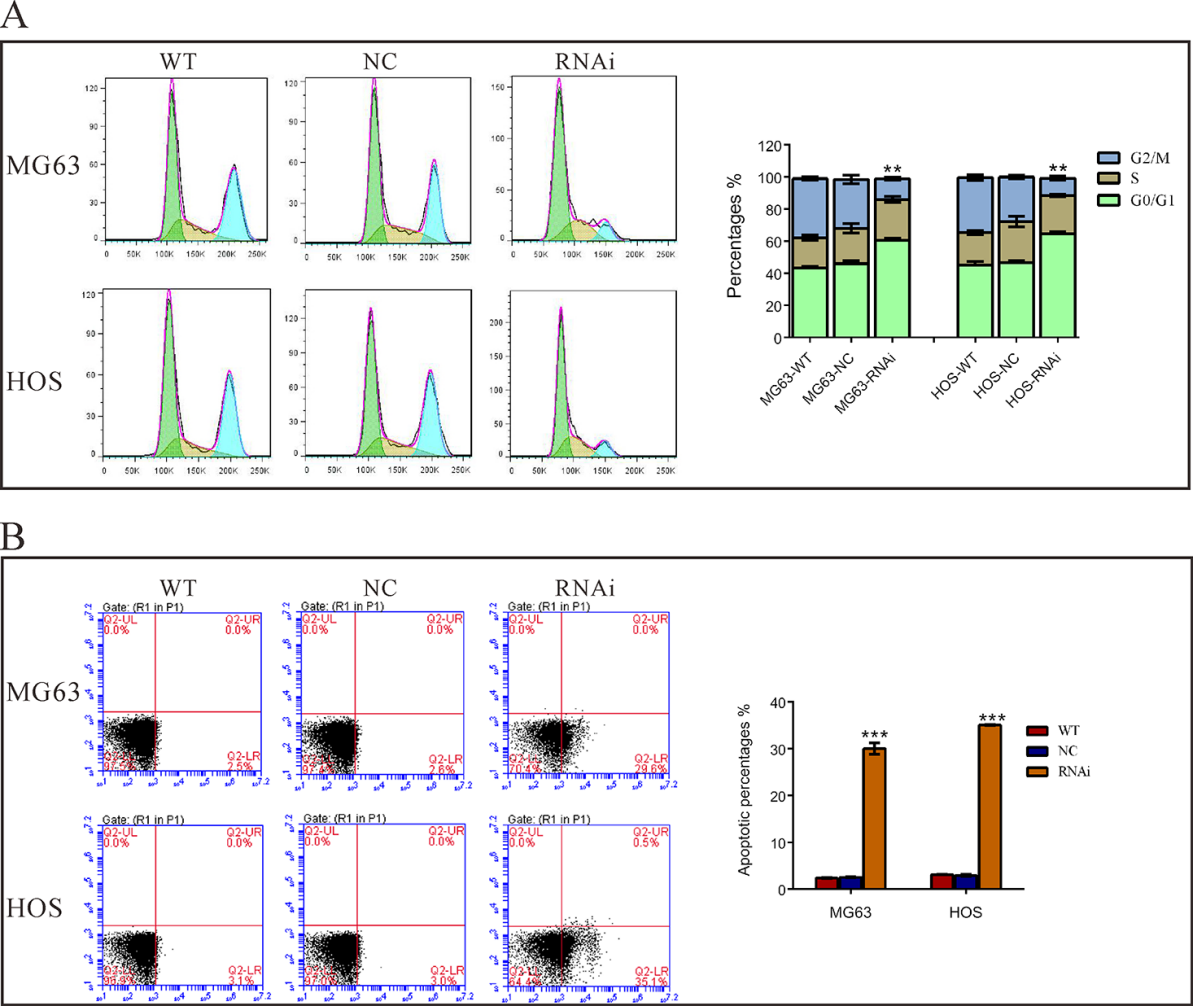
Silencing of TRIM66 induced G0/G1 arrest and cell apoptosis in osteosarcoma cells MG63 and HOS cells were transfected with indicated siRNA and 48 hours later cells were collected. **A.** Cell cycle profile was analyzed using flow cytometry; **B.** Cells were double stained with annexin V-FITC/PI and apoptosis rates was analyzed using flow cytometry. Data were based on at least three independent experiments, and shown as mean ± S.D. Representative images and quantitative results were shown. WT: wild type cells; NC: scrambled siRNA-transfected cells; RNAi: TRIM66-siRNA-transfected cells (**P < 0.01, ***P < 0.001 as compared with NC).

Then we assessed whether TRIM66 affects the cell cycle of osteosarcoma cells by PI staining and flow cytometry analysis. As shown in Figure 3A, compared with cells transfected with scramble siRNA, TRIM66-siRNA transfection caused a significant increase of G0/G1 phase cells (increased ratio: MG63, 39.1%; HOS, 32.0%) and a notable decrease of G2/M phase cells (decreased ratio: MG63, 57.8%; HOS: 60.6%).

We then explored the effects of TRIM66 in the apoptosis of osteosarcoma cells by Annexin V-FITC/PI staining assay. As shown in Figure 3B, 12-fold increase in cell apoptosis was noted in TRIM66-siRNA transfected MG63 and HOS cells as compared to scramble siRNA. These results indicated an anti-apoptotic role of TRIM66 in osteosarcoma.

### Silencing of TRIM66 inhibited the motility and invasiveness of osteosarcoma cells

**Figure 4:**
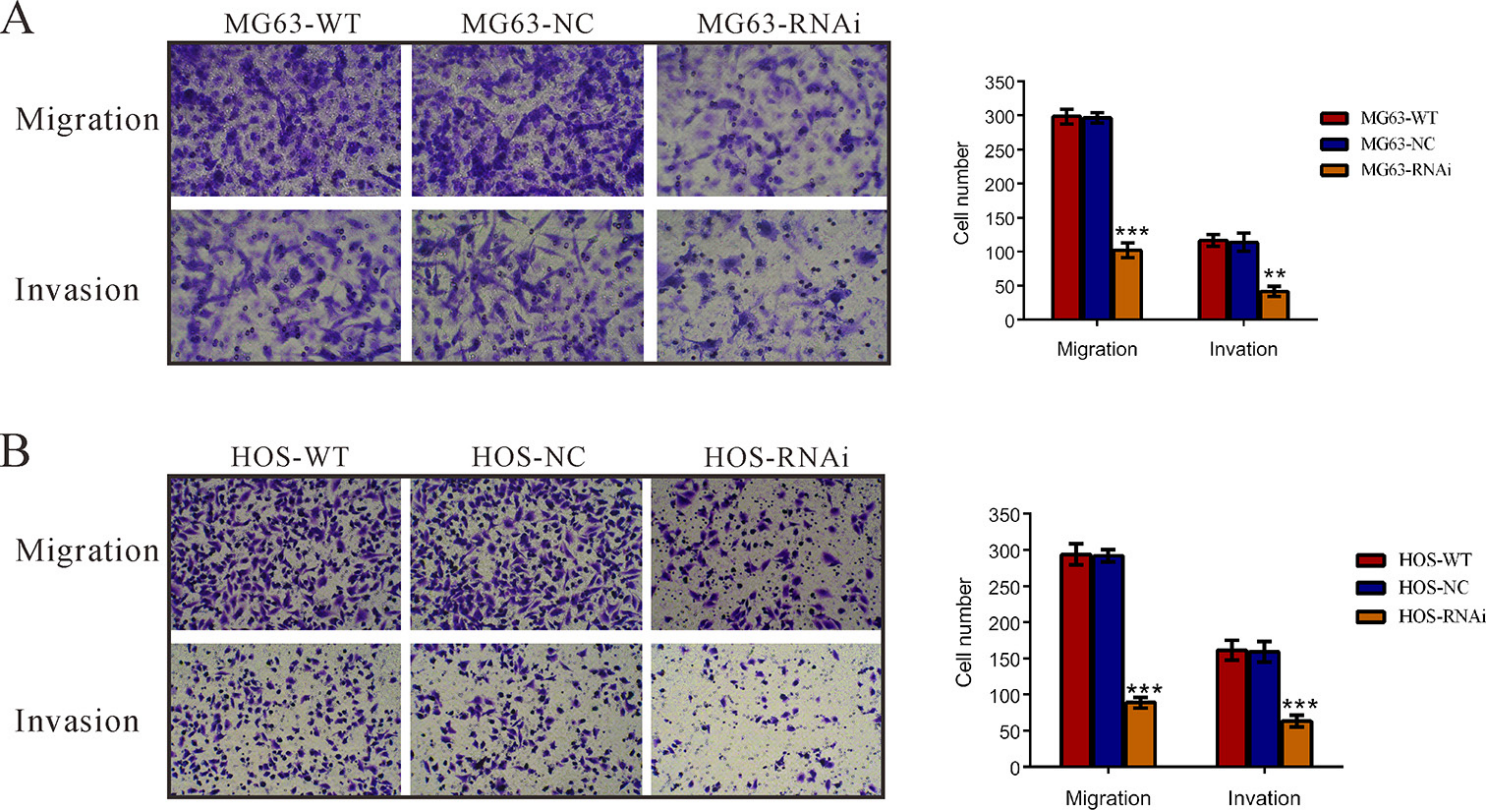
Silencing of TRIM66 inhibited cell migration and invasion in osteosarcoma cells MG63 and HOS cells transfected with indicated siRNA and cell migration and invasion was analyzed as described in Materials and Methods **A.** knockdown of TRIM66 notably inhibited cell migration and invasion. Data were based on at least three independent experiments, and shown as mean ± S.D. Representative images and quantitative results were shown. WT: wild type cells; NC: scrambled siRNA-transfected cells; RNAi: TRIM66-siRNA-transfected cells (**P < 0.01, ***P < 0.001 as compared with NC).

Then, we investigated whether TRIM66 affected the migrated and invasive ability of osteosarcoma cells. Suppressing of TRIM66 expression brought about a significant reduction in the migration of MG63 and HOS cells (Figure 4). Similar numbers of wild-type and scramble siRNA-transfected cells migrated to the lower face of the transwell membrane (MG63: WT, 294 ± 9; NC: 292 ± 5; HOS: WT, 298 ± 6; NC: 296 ± 4), whereas the TRIM66 knockdown cells exhibited a strongly inhibited motility, with only 30.5 and 34.5% cells migrating (MG63: RNAi, 89 ± 4; HOS: RNAi, 102 ± 7). Additionally, TRIM66 knockdown cells showed significant reduced invasive ability compared to control cells. The number of invaded cells was 36.8% and 39.6% of that of the control cells in MG63 and HOS cells, respectively (MG63: WT, 117 ± 5; NC, 114 ± 8; RNAi, 42 ± 4; HOS: WT, 161 ± 8; NC, 159 ± 8; RNAi, 63 ± 5). Moreover, the invasive ability of a lower-TRIM66 expression cells, U-20S, was increased by TRIM66 overexpression (Supplementary Figure S2). These data suggested a role of TRIM66 in the promotion of osteosarcoma metastasis.

### Knockdown of TRIM66 suppressed tumorigenicity of osteosarcoma cells in nude mice

**Figure 5:**
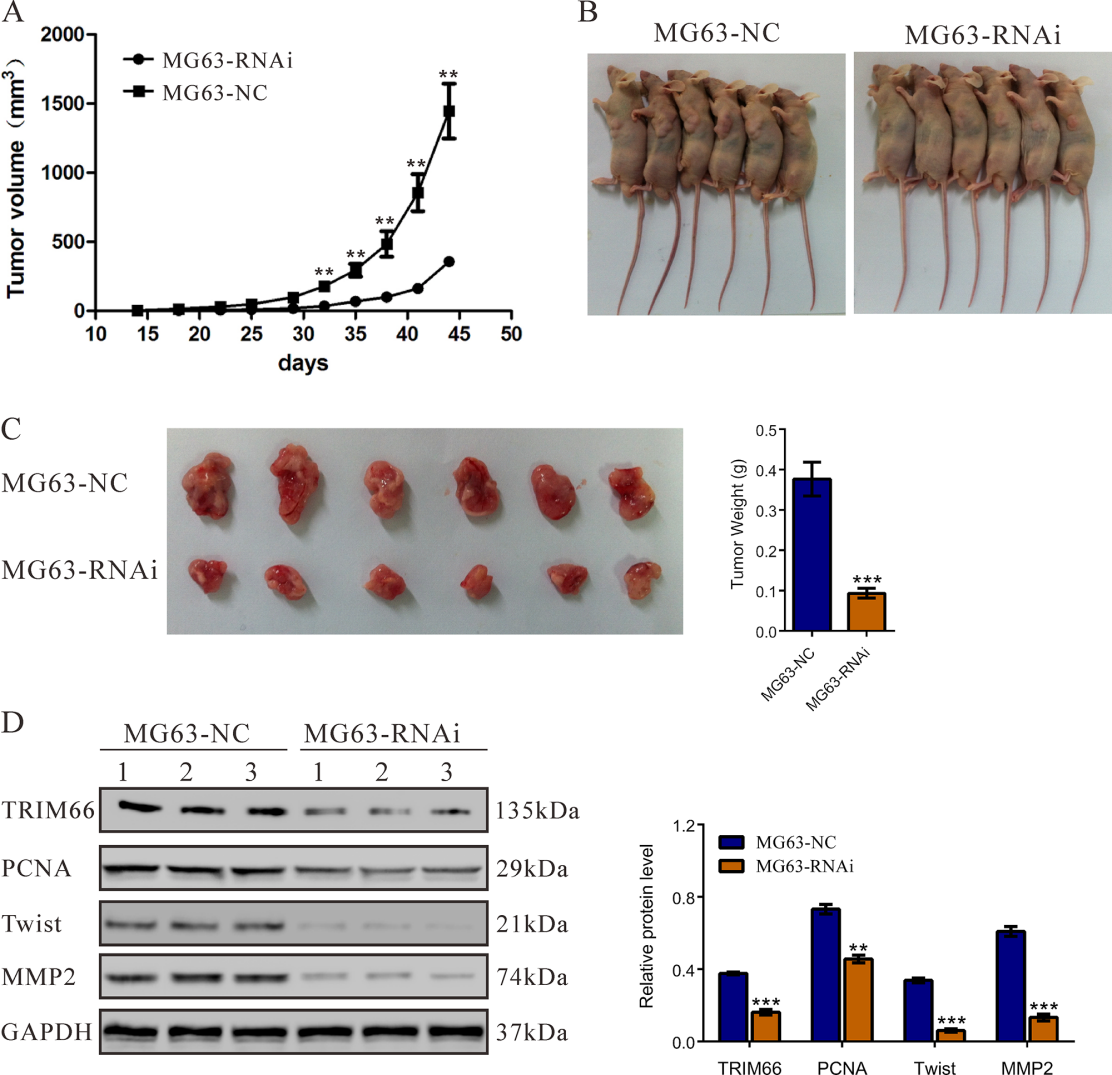
Knockdown of TRIM66 in osteosarcoma cells reduced tumor growth *in vivo* MG63 cells transfected with siRNA control (NC) or TRIM66-siRNA were subcutaneously injected in athymic nude mice. **A.** Tumor volume was evaluated for 45 days. Tumor growth was significantly slower in mice injected with TRIM66 knockdown cells. The mean value ± S.D. were from six animals in each group (**P < 0.001); **B.** Xenografts in nude mice at day 45; **C.** At day 45, mice were sacrificed and tumors were weighted. The six tumor tissues derived from the RNAi group were smaller than that from the control group; **D.** Western blot analysis of TRIM66, PCNA, Twist and MMP2 in xenografts from nude mice (**P < 0.01, ***P < 0.001 as compared with NC).

To determine the effect of TRIM66 on tumorigenicity *in vivo*, equal number of MG63 cells transfected with scramble siRNA or TRIM66-siRNA was injected subcutaneously into nude mice and tumor formation was examined for 45 days. As shown in Figure 5, although both cells were able to form tumors, the tumor growth rate of mice injected with TRIM66-siRNA cells was significantly slower than that of mice injected with control cells. The volume and weight of TRIM66-siRNA tumors was less than 25% that of control tumors. TRIM66 in TRIM66-siRNA tumors was significantly lower than that in control tumors. Further, the proliferation (PCNA) and invasion (MMP2 and Twist) related proteins were significantly decreased in tumors with TRIM66 knockdown compared with in NC control.

### Identification of genes and signaling associated biological pathways and processes by gene set enrichment analysis (GSEA)

**Figure 6:**
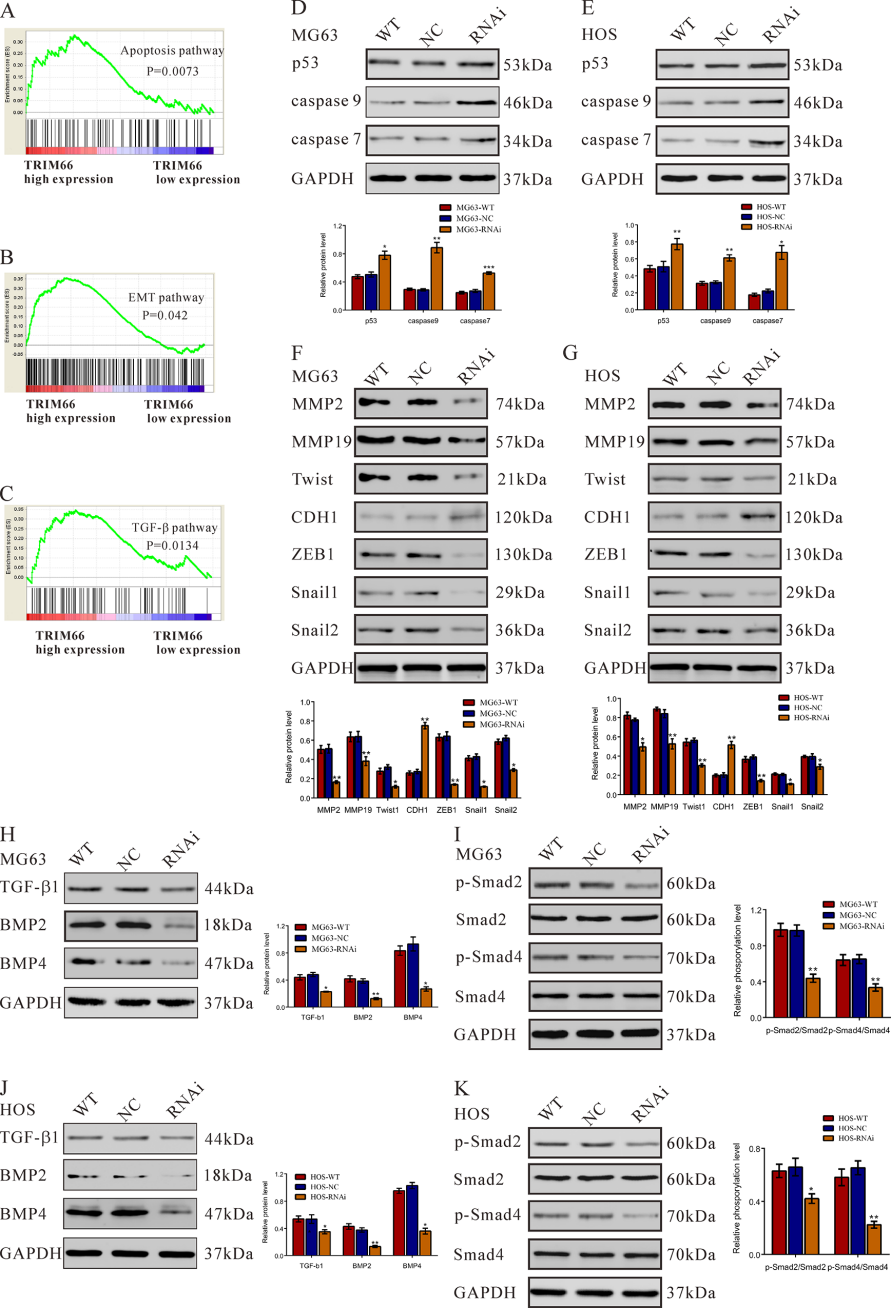
Apoptosis, EMT and TGF-β pathways were affected by TRIM66 siRNA treatment in osteosarcoma cells **A, B, C.** GSEA was performed using E-MEXP-3628 dataset. Apoptosis, EMT and TGF-β pathway were identified with the strongest association with TRIM66-higher expression. Expression of three apoptosis pathway related gene was evaluated by Western blot in **D.** MG63 and **E.** HOS cells transfected with TRIM66-siRNA or siRNA control (NC). Expression of EMT pathway related gene was evaluated by Western blot in **F.** MG63 and **G.** HOS cells. Expression of TGF-β pathway related gene and phosphorylation of Smad2 and Smad4 was evaluated by Western blot in **H, I.** MG63 and **J, K.** HOS cells transfected with TRIM66-siRNA or siRNA control (NC). Data were based on at least three independent experiments, and shown as mean ± S.D. Representative images and quantitative results were shown. WT: wild type cells; NC: scrambled siRNA-transfected cells; RNAi: TRIM66-siRNA-transfected cells (*P < 0.05, **P < 0.01, ***P < 0.001 as compared with NC).

To probe the TRIM66-associated pathways on an unbiased basis, we performed GSEA using data from the GEO E-MEXP-3628 dataset. GSEA is designed to detect coordinated differences in expression of predefined sets of functionally related genes. The apoptosis, EMT and TGF-β pathways were identified with the significant association with TRIM66 high expression in the E-MEXP-3628 dataset (Figure 6A–6C).

### TRIM66 siRNA treatment increased the protein levels of p53, caspase 7 and caspase 9 in osteosarcoma cells

After 48 h of TRIM66 siRNA treatment, the protein levels of p53, caspase 7 and caspase 9, which are closely related to apoptosis of tumor cells, were analyzed by Western blot. As revealed in Figure 6D and 6E, TRIM66 siRNA significantly increased the expression levels of p53 (increased ratio: MG63, 54.2%; HOS, 52.0%), caspase 9 (increased ratio: MG63, 207.9%; HOS, 88.7%) and caspase 7 (increased ratio: MG63, 54.2%; HOS, 204.3%).

### TRIM66 siRNA treatment regulated the expression of EMT pathway proteins in osteosarcoma cells

A few MG63 cells transfected with siRNA control (NC) changed from a classical epithelial morphology to spindle-type morphology, while no such change was observed in TRIM66 knockdown cells (Supplementary Figure S1). Further, the expression of EMT pathway proteins, which are closely related to metastasis of tumor cells, were also analyzed by Western blot. TRIM66 siRNA remarkably reduced the expression levels of MMP2 (decreased ratio: MG63, 67.9%; HOS, 25.5%), MMP19 (decreased ratio: MG63, 40.0%; HOS, 27.4%), Twist1 (decreased ratio: MG63, 63.4%; HOS, 46.3%), ZEB1(decreased ratio: MG63, 78.3%; HOS, 63.1%), Snail1 (decreased ratio: MG63, 72.6%; HOS, 47.1%) and Snail2(decreased ratio: MG63, 53.3%; HOS, 27.0%), while increased the main factor of EMT, E-cadherin (CDH1) (increased ratio: MG63, 173.9%; HOS, 152.3%).

### TRIM66 siRNA down-regulated TGF-β signaling

TRIM66 siRNA treatment significantly increased the expression levels of TGF-β (decreased ratio: MG63, 54.2%; HOS, 34.7%), BMP 2 (decreased ratio: MG63, 67.3%; HOS, 64.5%) and BMP 4 (decreased ratio: MG63, 71.2%; HOS, 65.0%).

After 6 h of TRIM66 siRNA treatment, the phosphorylation levels of Smad2 and Smad4, downstream of TGF-β signaling, were also analyzed by Western blot. As shown in Figure 6I and 6K, TRIM66 siRNA significantly decreased the relative phosphorylation levels of p-Smad2/Smad2 (decreased ratio: MG63, 54.9%; HOS, 48.7%) and p-Smad4/Smad4 (decreased ratio: MG63, 36.1%; HOS, 65.7%).

In order to further confirm the involvement of TGF-β signaling, U-20S was overexpressed with TRIM66 and treated with TGF-β inhibitor (SB431542, Supplementary Figure S2). The inhibitory effects of SB431542 on cell invasion in osteosarcoma cells was weakened by TRIM66 overexpression, which suggested TRIM66 may promote cell invasion partially by activating TGF-β pathway.

## DISCUSSION

Changes in several TRIM family protein have been implicated in several cancers [8, 9, 20, 21, 22, 23, 24]. However, the expression and functional implication of TRIM66 in cancers has been poorly defined. In the current study, we reported a significant elevation of TRIM66 in osteosarcoma tissues and its high expression was associated with poor prognosis of this disease. The *in vitro* experiments showed that silencing of TRIM66 expression in osteosarcoma cells inhibited cell growth, metastasis and tumorigenicity in nude mice, while induced cell apoptosis.

Firstly, our study identified TRIM66 as a useful biomarker for the diagnosis and prognosis of osteosarcoma (Figure 1). Our own real-time PCR results and analysis on an independent dataset demonstrated that TRIM66 mRNA level was higher in osteosarcoma tissues as comparing to bone cysts or normal bone tissues. Further, our immunohistochemistry results also demonstrated an increase of TRIM66 protein in 63.4% of osteosarcoma tissues. High expression level of TRIM66 was associated with local recurrence, lung metastasis (Table 1) as well as patient's prognosis (Figure 1D and 1E). These results indicated the possible clinical value of TRIM66 in osteosarcoma.

Then we examined the association of TRIM66 and osteosarcoma by knockdown its expression in two osteosarcoma cell lines. Here, we found that knockdown of TRIM66 significantly impaired cell growth (Figure 2) and cell cycle progression (Figure 3A) in osteosarcoma. Moreover, depletion of TRIM66 notably inhibited cell migration, cell invasion (Figure 4) and tumorigenicity in nude mice (Figure 5), while significantly induced cell apoptosis (Figure 3B). These data demonstrated the association of TRIM66 with the carcinogenesis of osteosarcoma.

The exact pathway that TRIM66 may regulate in osteosarcoma remains unclear. Further, we performed GSEA on E-MEXP-3628 dataset and identify that TRIM66 expression was positively correlated with several cancer-related networks, including apoptosis, EMT and TGF-β pathway, which was then confirmed in osteosarcoma cells by Western blot (Figure 6).

p53 is the most commonly tumor suppressor gene in human cancer. p53-dependent apoptosis contributes to the tumor suppressor activity and chemotherapy-induced cell death [25]. The importance of caspase 9 and caspase 7 in p53-dependent apoptosis has been extensively explored [26]. Recent studies have demonstrated that TRIM28 and TRIM24 contributes to p53 inactivation. TRIM24 ubiquitinates p53 both *in vivo* and *in vitro* via its RING domain [27]. TRIM28 interacts with several proteins, including Mdm2 and E2F1, resulting in indirect effects on p53 stability [28, 29]. Here, a significant increase of p53, caspase 9 and caspase 7 was observed in TRIM66-siRNA-transfected cells (Figure 6D & 6E). Our data suggested that TRIM66 may inhibit osteosarcoma cell apoptosis through down-regulating p53. Whether TRIM66 directly or indirectly regulates p53 activity needs to be further investigation.

Osteosarcoma is characterized by a highly malignant and metastatic potential. The Epithelial-mesenchymal transition (EMT) is involved in complex pathogenesis of tumors [30, 31]. Here, TRIM66 siRNA treatment stimulated the expression of the main factor of EMT (E-cadherin, CDH1), but decreased the expression of five known inducers of EMT (β-catenin, ZEB1 [32], Twist, Snail1 and Snail 2 [33]) (Figure 6F & 6G). Our data suggested that TRIM66 siRNA may inhibit osteosarcoma cell invasion through suppressing EMT.

Perturbations of TGF-β signaling are central to tumorigenesis and tumor progression by affecting cell proliferation, invasion and EMT [34]. TRIM33 (TIF1γ), a member of TRIM family, was recently identified as a Smad4-independent regulator of TGF-βsignaling [35]. Here, we found that silencing of TRIM66 expression significantly decreased the protein levels of TGF-β1, BMP2 and BMP4 and impaired the TGF-β signaling pathway (Figure 6H–6K). Further, the inhibitory effects of TGF-β inhibitor, SB431542 on cell invasion in osteosarcoma cells was weakened by TRIM66 overexpression (Supplementary Figure S2), which suggested TRIM66 may promote cell invasion partially by activating TGF-β pathway.

In summary, our study provides for the first time that TRIM66 promoted the proliferation and metastasis via TGF-β signaling pathway, and inhibited cell apoptosis via down-regulating p53 expression in osteosarcoma cells. As TRIM66 expression level associated with patients’ survival rate, TRIM66 might be a useful prognostic factor for osteosarcoma.

## MATERIALS AND METHODS

### The patients and tissue samples

101 patients with osteosarcoma and 14 patients with bone cysts who were treated at Department of Orthopedic Surgery, Changzheng Hospital (Shanghai, China) between 2000 and 2006 were enrolled in this study. The clinicopathologic variables such as gender, age, the site of osteosarcoma, the stage and recurrence were retrospectively reviewed on the basis of the medical records. Informed and written consent was obtained from all patients or their advisers. The study was approved by the independent ethics committee of Changzheng Hospital, Second Military Medical University.

### Immunohistochemistry

Immunohistochemical (IHC) staining was performed on the formalin-fixed, paraffin-embedded surgical specimens from all the patients. The sections were deparaffinized, rehydrated and then processed using the labeled streptavidin-biotin-peroxidase method. Antigen retrieval was performed by microwave heating for 5 minutes in 0.1 mol/L citrate buffer (pH 6.0) followed by blocking of endogenous peroxide with 3% H_2_O_2_ in methanol for 5 minutes. After PBS washes, slides were blocked with 10% normal blocking serum for 30 minutes. After that, they were incubated with antibody against TRIM66 (Abcam) overnight at 4°C, and then incubated with biotin-labelled secondary antibodies at room temperature for 1 hour. The slides were reacted with the ABC kit and DAB substrate (Vector Laboratory), and hematoxylin was used as the nuclear counterstain. The TRIM66 expression was graded as negative (no positive stained), low (< 25% of the cells stained) and high (> 25% of the cells stained). The assessment of the staining was conducted by two investigators independently.

### Cell culture

MG63, HOS, U-20S, Saos2 and SW1353 cells were purchased from cell bank of Shanghai biology institute, Chinese Academy of Science (Shanghai, China). All culture media were supplemented with 10% fetal bovine serum (FBS, Life Technologies), 2 mM L-glutamine and 1% penicillin/streptomycin (Life Technologies). MG63, HOS, Saos2 and SW1353 cells were grown in DMEM Medium (life technology). U-20S cells was grown in RPMI 1640 medium (life technology). All of the cell lines were maintained at 37°C in a 5% CO_2_ atmosphere.

### RNA interference

One siRNA targeting position 6400-6420 (TACGGTGAGTTCTAGATTCTT; named TRIM66-siRNA) of human TRIM66 mRNA (NM_014818.1) were synthesized. A non-specific scramble siRNA sequence was used as negative control (NC). The siRNAs were transiently transfected into MG63 and HOS cells using Lipofectamine 2000 (Invitrogen) according to the manufacture's instruction. Assays were performed 48 h after transfection.

### Reverse transcription and real-time PCR

Total RNA was extracted by TRIzol Reagent (Invitrogen) and reverse transcribed by cDNA synthesis kit (Thermo Fisher) according to the manufacturer's instructions. Quantitative PCR was performed with SYBR Green qPCR Master Mixes (Thermo Fisher) on an ABI 7300 system (Applied Biosystem) using the following cycling parameters, 95°C for 10 min, followed by 40 cycles of 95°C for 15 s, 60°C for 45 s. GAPDH was served as an internal control. The gene expression was calculated using the Δ;Δ Ct method. All data represent the average of three replicates. The primers used were list as follows:

TRIM66 (NM_014818.1): forward primer 5′-GCCCTCTGTGCTACTTACTC-3′, reverse primer 5′-GCTGGTTGTGGGTTACTCTC-3′;

GAPDH (NM_001256799.1): forward primer 5′-CACCCACTCCTCCACCTTTG-3′, reverse primer 5′-CCACCACCCTGTTGCTGTAG-3′.

### Antibodies and immunoblotting

Primary antibodies were obtained from the following companies: (1) TRIM66, PCNA, caspase7, caspase9, MMP2, MMP19, Twist11, CDH1, Snail1, Snail2, ZEB1 and TGF-β1, Abcam; (2) p-Smad4, Thermo Fisher; (3) p53, p-Smad2, Smad2, Smad4 and GAPDH, CST Biotech. Horseradish peroxidase-conjugated goat anti-mouse secondary antibody or goat anti-rabbit secondary antibody was purchased from Beyotime.

Treated and untreated cells were lysed in RIPA lysis buffer with fresh added protease inhibitor cocktail (Sigma). Protein concentration was assayed using BCA protein assay (Thermo Fisher). Equal amounts of protein were then subjected to SDS gel electrophoresis. Following SDS-PAGE, proteins were transferred to a nitrocellulose membrane and immunoblotted with the respective antibodies. Signals were developed by enhanced chemiluminescence (ECL, Millipore). Band intensities were measured using Image J (NIH) and normalized to GAPDH.

### Cell proliferation assay

Cell proliferation was measured using Cell Counting Kit-8 CCK-8 Assay Kit (Dojindo Lab, Kumamoto, Japan) according to manufacturer's protocol. Briefly, treated and untreated HOS and MG63 cells were seeded in 96-well plates. At indicated time point, CCK8 solution (10 μl in 100 μl DMEM medium) was added to each well and incubated for 1 h. Optical density values (OD) at wavelength 450 nm was measured by a microplate reader (Bio-Rad).

### Cell cycle distribution analysis

Propidium iodide (PI) staining was used to analyze DNA content. Treated and untreated cells were harvested, resuspended in PBS and fixed with 70% ethanol at −20°C for at least 2 hours. After treatment with ribonuclease (Sigma) for 15 min at 37°C, PI (0.05 mg/ml, Sigma) was added to the cells, followed by incubation at room temperature in the dark for 30 min. DNA content was then analyzed on a flow cytometer (BD Biosciences). The percentage of cells in the G0/G1, S, and G2/M phases was determined by the FlowJo cell cycle analysis program.

### Cell apoptosis assay

The percentage of cells actively undergoing apoptosis was determined by double stained with Annexin V-fluorescein isothiocyanate (FITC) and PI. Both adherent and floating cells were harvested, incubated with Annexin V-FITC and PI (BD Biosciences), and then analyzed by flow cytometry (BD Biosciences). At least 20,000 cells were acquired for each sample.

### Migration assay

Cell migration assays were performed using Boyden chambers (Coring Incorporated) as described previously [16]. Briefly, cells transfected with indicated siRNA were serum starved for 24 h and then seeded in the upper chamber, while the medium supplemented with 30% fetal bovine serum was placed in the lower chamber. After 24 h of incubation, cells on the upper side of the filters were removed and the remaining cells were fixed in 4% formaldehyde and stained with 0.05% crystal violet. Finally, the cells that had migrated to the lower surface of the membrane were stained with crystal violet and counted under the microscope.

### Invasion assay

Cell invasion assays were also performed in Boyden chambers containing a polycarbonate filter coated with Matrigel on the upper surface. The rest of the assay was performed as described above.

### Xenograft model

The experiment was performed under the Institute's guidelines for animal experiments. For each cell line, 2 × 10^6^ cells were subcutaneously injected into the right flank of 4–6-week-old BALB/c nude mice (Shanghai Laboratory Animal Company). Tumor length and width were measured every 3 days after injection. Tumor volume was calculated as length × (width^2^/2).

### Gene set enrichment analysis (GSEA)

GSEA is a method of analyzing and interpreting microarray and such data using biological knowledge [17]. In this study, GEO E-MEXP-3628 dataset was analyzed by GSEA as previously described [16, 18, 19]. GSEA firstly generated an ordered list of all genes according to their correlation with TRIM66 expression, and then a predefined gene set (signature of gene expression upon perturbation of certain cancer-related gene) receives an enrichment score (ES), which is a measure of statistical evidence rejecting the null hypothesis that its members are randomly distributed in the ordered list. The expression level of TRIM66 gene was used as phenotype label, and “Metric for ranking genes” was set to Pearson Correlation.

### Statistical analysis

The Statistical Package for Social Sciences (SPSS) version 17.0 for Windows (SPSS, Inc., Chicago, IL) was used for the statistical analysis. The Chi-square tests were used to compare the clinicopathologic characteristics of tumors (and patients) with high and low TRIM66 expression. For survival analysis, overall survival was defined as the time interval between the date of surgery to the date of death or the last follow-up. Survival curves were calculated using the Kaplan-Meier method, and comparisons between the different groups were analyzed using the log-rank test. The two-tailed Student's *t*-test was used to evaluate statistical differences between two groups. The accepted level of statistical significance was *P* < 0.05.

## SUPPLEMENTARY FIGURES


