# Supplementary material for: TRIM66 overexpresssion contributes to osteosarcoma carcinogenesis and indicates poor survival outcome
Source: Oncotarget. 2015 Jun 17;6(27):23708–19. doi: 10.18632/oncotarget.4291 (PMC4695146; doi:10.18632/oncotarget.4291)
Supplement: Supplementary file 1 [file oncotarget-06-23708-s001.pdf]

SUPPLEMENTARY FIGURES

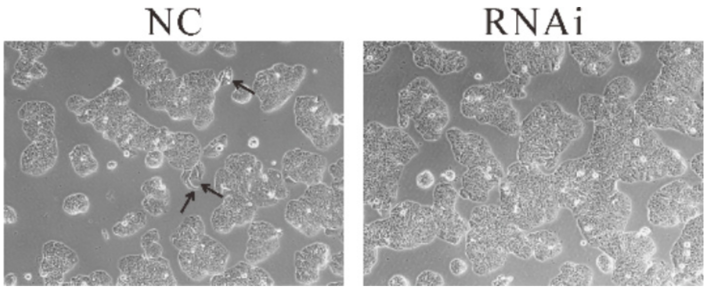

Supplementary Figure S1: Phase contrast images of MG63 cells transfected with siRNA control (NC) or TRIM66-siRNA. A few MG63 cells in NC group showed spindle-type morphology (indicated by arrow). Magnification, × 100.

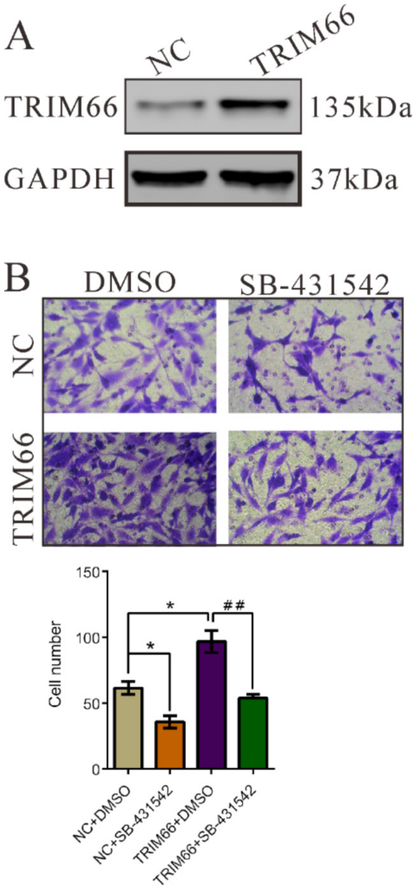

Supplementary Figure S2: TRIM66 overexpression reversed the effects of TGF-β inhibitor on cell invasion of U-20S. A. U-20S cells was transfected with PWPXL-TRIM66 or control plasmid (NC). Expression of TRIM66 was analyzed by western blot. **B.** U-20S cells transfected with PWPXL-TRIM66 or control plasmid were trypsinized and placed onto Matrigel-coated filters in a Boyden chamber containing either DMSO or 10 µm TGF-β inhibitor, SB431542 (Sigma). Cells were allowed to migrate for 24 hours then were fixed and stained (*$P < 0.05$ as compared with NC+DMSO, ##$P < 0.01$ as compared with TRIM66 + DMSO).
